# Reported Tuberculosis Symptoms: An Inadequate Classifier of Disease State

**DOI:** 10.1093/cid/ciaf611

**Published:** 2025-12-10

**Authors:** Nicky McCreesh, Peter MacPherson, José Victor Bortolotto Bampi, Nora Engel, Katharina Kranzer, Palwasha Y Khan

**Affiliations:** TB Centre, London School of Hygiene & Tropical Medicine, London, United Kingdom; TB Centre, London School of Hygiene & Tropical Medicine, London, United Kingdom; Public Health Group, Malawi Liverpool Wellcome Programme, Blantyre, Malawi; School of Health & Wellbeing, University of Glasgow, Glasgow, United Kingdom; Faculty of Medicine, Federal University of Mato Grosso do Sul, Campo Grande, Brazil; Athena Institute, Faculty of Science, Vrije Universiteit Amsterdam, Amsterdam, The Netherlands; TB Centre, London School of Hygiene & Tropical Medicine, London, United Kingdom; The Health Research Unit Zimbabwe, Biomedical Research and Training Institute, Harare, Zimbabwe; Institute of Infectious Diseases and Tropical Medicine, LMU University Hospital, LMU Munich, Munich, Germany; TB Centre, London School of Hygiene & Tropical Medicine, London, United Kingdom

**Keywords:** tuberculosis, asymptomatic, subclinical

## Abstract

Around half of people identified with tuberculosis (TB) during prevalence surveys do not report symptoms, prompting increased interest in the public health implications of asymptomatic TB (aTB), as distinct from symptomatic TB (sTB). A recent World Health Organization report proposes investigating the optimal treatment for aTB, stratifying notification data by symptom status, and publishing incidence estimates based on sTB. We have concerns that the proposed use of aTB/sTB case definitions for surveillance, burden estimation, and clinical management is not supported by evidence. Although TB symptoms are linked to disease severity, we show that self-reported symptoms are an inadequate classifier of disease state, heavily influenced by context and substantial interviewer variability, and TB symptoms may frequently have alternative causes. We advocate for the adoption of more robust measures of severity, better reflecting underlying pathophysiology. Research on the TB disease spectrum should prioritize more objective severity metrics, alongside or instead of symptom-based classification.

## Teaser

We provide evidence that self-reported symptoms are an inadequate classifier of disease state, heavily influenced by context and substantial interviewer variability. They should therefore not be used as the primary criteria to determine treatment or to inform burden estimation.

## BACKGROUND

How we categorize tuberculosis (TB) has evolved over time, as the primary methods used to detect it have changed. In the mid-20th century, many (predominantly industrialized) countries made use of mass chest X-ray screening, identifying a broad spectrum of TB disease, including individuals without noticeable symptoms [1]. As rates of TB fell in high-income countries during the second half of the 20th century, global TB prevention and care efforts shifted toward low- and middle-income countries. In these settings, resource constraints led to a focus on diagnosing and treating symptomatic infectious (at the time, equated with smear-positive) TB, reinforcing the idea that symptoms define disease [1].

With global TB incidence declining by only 1% per year [2], recent years have seen the pendulum swing back toward more intensive approaches to TB care and prevention, and a goal of achieving early diagnosis of all people with TB. This coincides with a focus on the considerable proportion of people with prevalent bacteriologically positive TB who do not report symptoms, and who may constitute an important reservoir of infectious TB, fueling ongoing transmission. Alongside this shift in focus, the detection of TB in people who do not report symptoms has become more feasible and affordable, thanks to technological developments such as digital radiography systems with computer-aided detection (CAD) software, and the broader availability of molecular diagnostics such as Xpert MTB/RIF.

In response to the growing interest in TB without reported symptoms, the WHO published a 2025 report on asymptomatic TB [3], following a technical consultation. The report introduces a distinction into symptomatic TB (sTB) and asymptomatic TB (aTB), based solely on whether individuals reported TB symptoms during screening. It also outlines a research and programmatic agenda around aTB, which can be broadly divided into 2 areas.

The first is around the impact of symptom-based versus symptom-agnostic screening. Here the division into aTB and sTB is clearly meaningful—the potential yields of symptom-based screening are inherently constrained by the proportion of people with TB who report symptoms during screening.

The second area goes much further, implicitly treating aTB and sTB as distinct and broadly meaningful disease states, with an importance that extends beyond their relevance to a specific occurrence of screening. This includes proposals to investigate the optimal treatment for aTB, stratify notification data by symptom status, and publish burden estimates that distinguish between aTB and sTB. In this commentary, we focus on this second area, presenting evidence that reported symptoms during screening have substantial limitations as indicators of disease state. We contend that in the majority of situations, more informative classifications would better reflect TB pathophysiology and have greater utility for person-centered clinical care and public health.

## TO WHAT EXTENT ARE “ASYMPTOMATIC TUBERCULOSIS” AND “SYMPTOMATIC TUBERCULOSIS” MEANINGFUL CASE DEFINITIONS?

Reported TB symptoms are undoubtedly correlated with TB severity, for instance, with a review of national TB prevalence surveys showing a moderate association between reported cough and sputum smear positivity [4]. More severe and/or persistent symptoms may provide a stronger indication of TB severity, compared to milder symptoms such as transient cough. In particular, there is some evidence to suggest that symptoms sufficient to prompt care seeking and passive diagnosis may be a more meaningful marker of severity than symptoms reported during active screening [5]. However, a statistical association is insufficient to justify the use of reported symptoms as a tool for disease classification—a usage that requires a robust, stable, and clinically/epidemiologically meaningful distinction between states. In this commentary, we provide evidence that reported symptoms during screening have substantial limitations as an indicator of disease state, both due to inconsistency of symptom reporting (eg between contexts or interviewers) and to imperfect associations between symptoms and disease state (eg due to symptoms with alternative causes).

### Inconsistency of Symptom Reporting by Setting, Interviewer, and Context

Self-reporting of TB symptoms is subjective and influenced by multiple contextual factors, including how, where, and by whom questions are asked [6]. The interpretation of symptoms—such as cough or night sweats—can vary significantly across settings, and people may avoid reporting symptoms to protect themselves from stigma [7]. Symptom reporting may also differ according to demographic characteristics, such as sex or socioeconomic status, shaped by prevailing norms. Moreover, reporting practices are not static; they can evolve over time. For example, the coronavirus disease 2019 (COVID-19) pandemic may have shifted perceptions of mild symptoms such as cough and altered the implications of disclosing—or withholding—such information.

**Figure 1.**
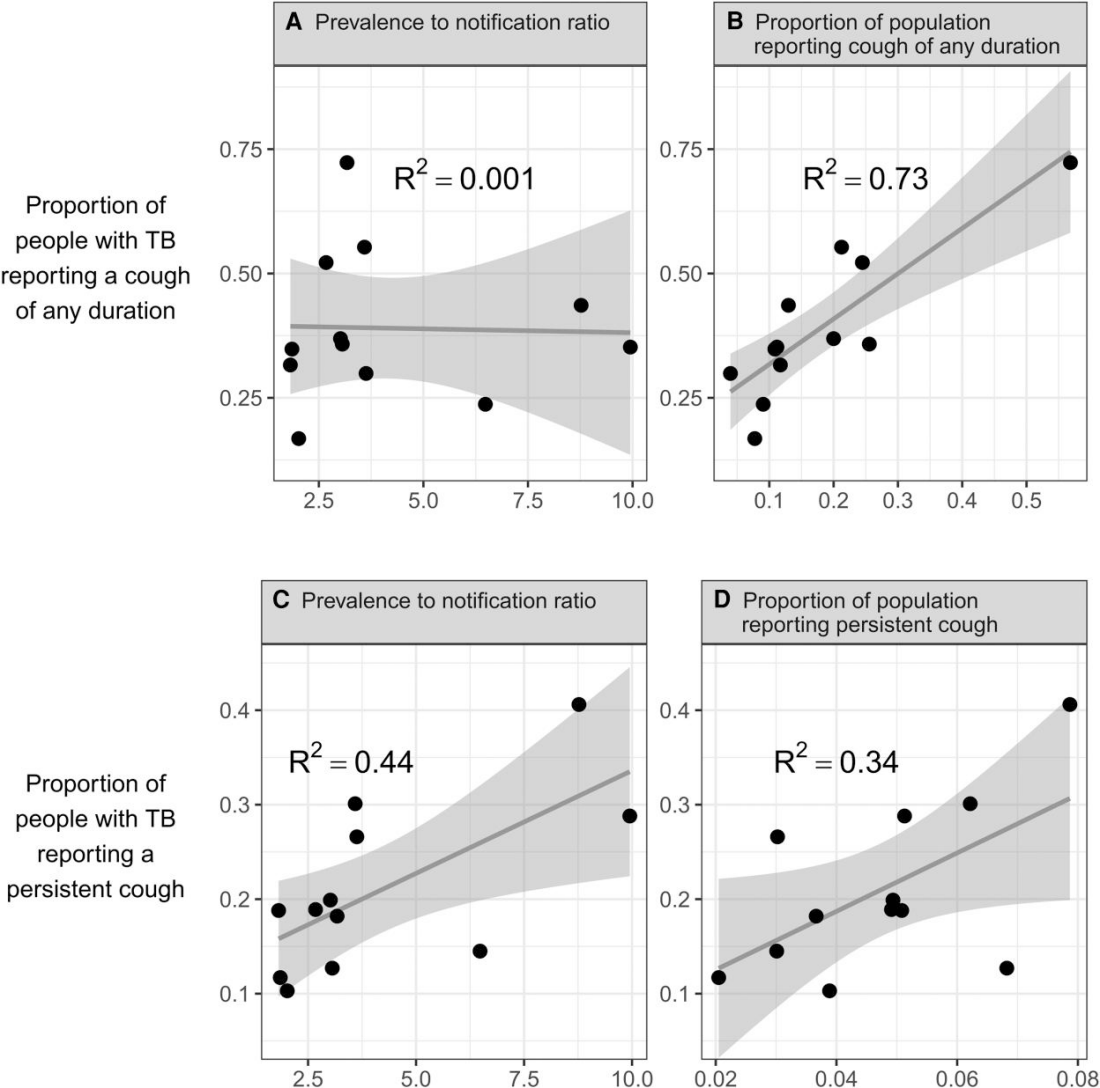
Relationship between the prevalence to notification ratio (*A* and *C*) and prevalence of reported cough (*B*) or persistent cough (*D*) in all participants, and the estimated proportion of people with TB reporting a cough of any duration (*A* and *B*) or persistent cough (*C* and *D*), in 12 national TB prevalence surveys. The estimated proportions of people with TB reporting cough of any duration or persistent cough are adjusted for false-negative chest X-rays and uninterpretable culture results, taken from Stuck *et al* (2024) [4]. Using unadjusted estimates gives a) *R*^2^ = 0.03, b) *R*^2^ = 0.39, c) *R*^2^ = 0.35, and d) *R*^2^ = 0.34. Abbreviation: TB, tuberculosis.

Data from national TB prevalence surveys and notification systems highlight the role of country context in symptom reporting. If symptoms reliably indicate more advanced TB disease, their prevalence should correlate strongly with the national prevalence-to-notification ratio, which reflects diagnostic delays and/or low treatment coverage. Yet across 12 countries with detailed symptom data, no such relationship is observed for cough of any duration (Figure 1*A*). Instead, there is a strong correlation between the proportion of people *with* and *without* TB reporting cough (Figure 1*B*), suggesting that country-specific factors substantially influence symptom reporting. *Persistent* cough appears to be a more robust indicator of disease state, as evidenced by a strong association with prevalence-to-notification ratios (Figure 1*C*). However, its reporting also closely follows general population trends (Figure 1*D*), underscoring the influence of setting and situation-specific factors.

**Figure 2.**
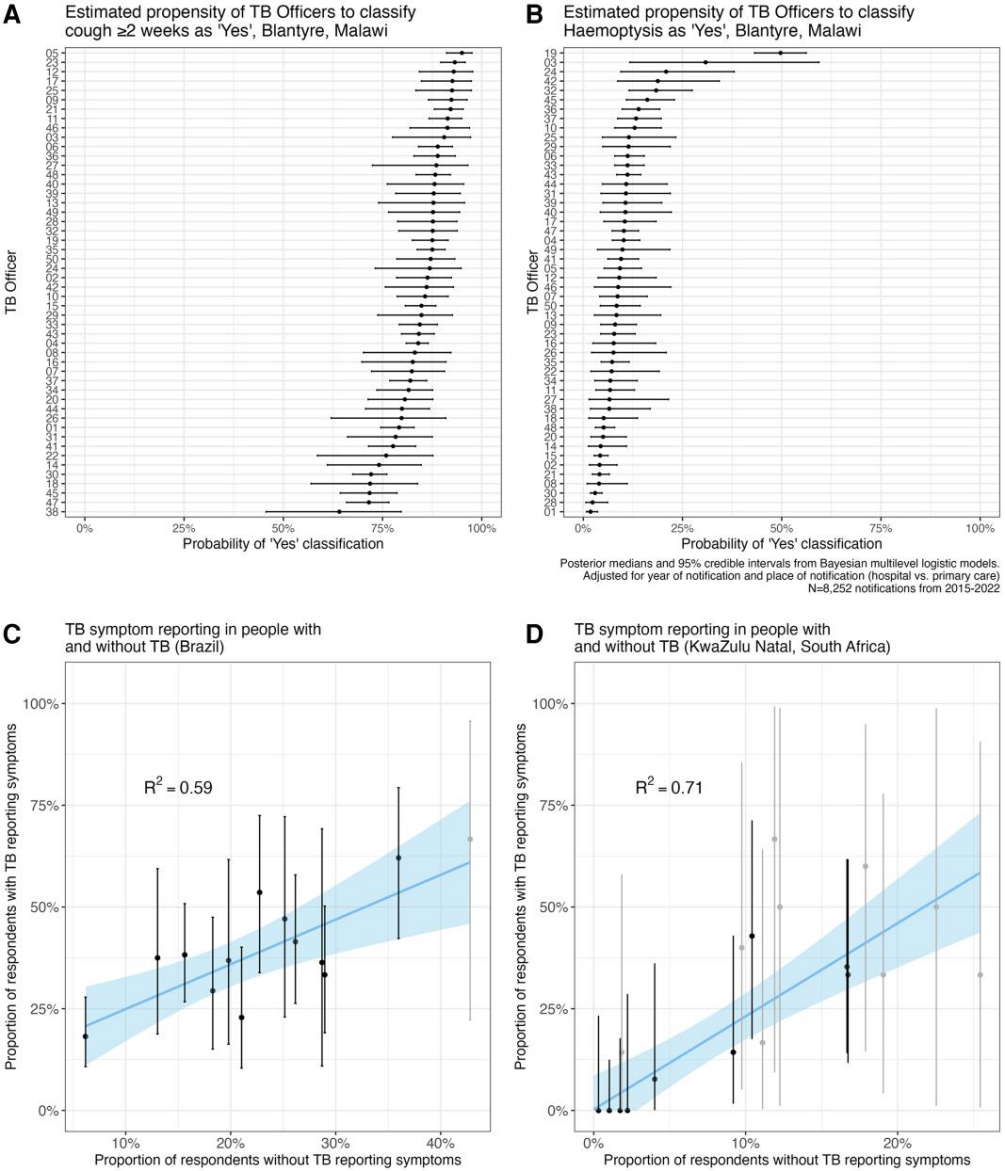
Variation in symptom reporting by interviewer. Estimated propensity of TB Officers to classify people diagnosed with TB in Blantyre, Malawi, in 2015–2022 as (*A*) having a cough lasting ≥2 wks and (*B*) hemoptysis. *C* and *D*, Relationship between the proportion of people without and with TB who reported symptoms during screening, by interviewer, in (*C*) prison-based screening in Brazil and (*D*) a community prevalence survey in South Africa. Tuberculosis symptoms are defined as one or more of cough of any duration, sputum, fever, appetite loss, night sweats, chest pain, trouble breathing, and weight loss of any duration in the Brazil study and one or more of cough of any duration, fever, night sweats, or weight loss in the South Africa study. Interviewers who interviewed fewer than 100 people in total were excluded. Data were weighted by the number of people with TB interviewed by the interviewer. Black and gray points and error bars indicate interviewers who interviewed 10 or more and fewer than 10 respondents with TB, respectively. Abbreviation: TB, tuberculosis.

Even within a single setting, the reporting of symptoms can vary markedly between interviewers. Figure 2*A* and 2*B*, based on TB notification data from Blantyre, Malawi (2015–2022) [8], shows wide variation in the proportion of individuals diagnosed with TB who reported a persistent cough or hemoptysis, depending on which TB officer conducted the interview. Figure 2*C* and 2*D* presents data from a study of TB case finding study in Brazilian prisons [9], and a TB prevalence survey conducted in KwaZulu-Natal, South Africa [10], demonstrating a strong correlation, by interviewer, between the proportion of individuals *without* and *with* TB who reported symptoms during screening—with each 10% absolute increase in the former associated with a 11% (Brazil) and 23% (South Africa) absolute increase in the latter. These data suggest that, even in research studies with extensive standardization and interviewer training, classification into aTB versus sTB may often depend as much on *who* conducted the interview than on the actual severity of the disease.

Finally, the extent to which individuals recognize and report symptoms is likely to vary according to the context in which they are asked. When asked about symptoms at screening, only 35% of people diagnosed with TB as part of the ACT3 community active case finding trial reported symptoms [11]. When asked again following diagnosis, a median of 7 days later, that proportion had more than doubled to 82%. Even for more objective symptoms such as weight loss, recognition and reporting may be low—among household contacts aged ≥10 years in Zimbabwe, Tanzania, and Mozambique who developed incident TB, only 8/22 (36%) with weight loss and 4/7 (57%) with substantial weight loss (>10% body mass index (BMI)) reported weight loss as a symptom [12]. More broadly, many clinicians encounter patients who initially do not report any symptoms, later acknowledging having experienced mild symptoms when questioned further. This not only undermines the reliability of symptom reporting but also poses a conceptual problem for defining aTB. A person's classification may shift from asymptomatic to symptomatic simply as a result of the effort to uncover their symptoms or retrospectively following diagnosis.

Taken together, these factors combine to mean that the measurement of TB symptoms is extremely unreliable, subject to large inter- and intraindividual variation, and is highly subject to person, setting, and cultural context factors.

### Alternative Causes of Tuberculosis Symptoms

Even in people diagnosed with TB, symptoms reported at the time of TB screening are not always caused by TB. Tuberculosis symptoms are nonspecific and commonly occur in individuals with other respiratory conditions, including bronchiectasis, pneumonia, respiratory viruses, and chronic obstructive pulmonary disease. People with chronic lung disease are also at increased risk of TB, making it difficult to disentangle which symptoms are attributable to TB versus underlying or coexisting conditions. In some cases, respiratory infections such as influenza may lead individuals with prior aTB to seek care, resulting in a diagnosis of sTB.

Findings from 32 national TB prevalence surveys conducted between 2007 and 2019 give an indication of the potential magnitude of incidental TB symptoms. Across these surveys, a median of 6.1% (range: 2.2%–21%) of all participants reported TB symptoms. Using data from the surveys on the proportions of all respondents and people with TB who reported symptoms, and assuming that the background prevalence of symptoms is the same in both groups, we estimate that a median of 8.2% (range: 2.5%–28%) of people with sTB do not have any TB symptoms *caused* by TB. Analysis of adjusted estimates from 12 surveys where more detailed symptom data are available [4] indicate that the estimate increases to 18% (range: 9%–50%) for persistent cough and 29% (range: 10%–62%) for cough of any duration. These figures may be underestimates, due to the increased risk of TB in people with other respiratory conditions.

## PROPOSED APPROACH

As we outline above, reported symptoms, while associated with disease severity, do not provide a robust, reliable, or generalizable indication of disease state. Despite these limitations, symptom status is an attractive way of classifying TB severity, due to its low cost, simplicity, and ease of implication at scale. Alternative and more robust metrics to classify disease state will frequently be available however, for instance, the quantification of sputum bacillary burden using molecular diagnostics or the extent and morphology of lung damage visible on chest X-ray (based on radiologist reading or CAD output). This is particularly true in contexts in which aTB is being diagnosed. It should also be noted that attempts to improve the reliability and generalizability of reported symptoms, for instance, interviewer training tailored to local contexts, will come with increased costs.

The WHO report raises questions around the optimization of treatment for aTB. We welcome the development of shorter and/or more tolerable regimens and a more nuanced approach to TB treatment. Given the limitations of reported symptoms as an indicator of disease severity, however, we advocate that symptoms (and less severe symptoms in particular) should not be the primary criteria for determining who receives less intensive regimens. Instead, more robust and objective metrics, such as those proposed in existing research on risk stratification [13], are likely to provide a better indication of people for whom a less intensive regimen is sufficient. This approach would also improve the applicability of treatment guidelines across different settings and risk groups, where symptom reporting may vary substantially.

Collecting data on reported symptoms at the time of screening will require extensive changes to data collection systems in most countries, with data currently collected following diagnosis only. Given the considerable burden involved in setting up the required systems and in collecting the addition data, it is important to consider whether the data are likely to be suitable for their intended purposes, in light of the substantial limitations of reported symptoms. If countries have the capacity to collect additional notification data, we would advocate for the addition of more objective measures of disease severity, such as Xpert semiquantitative category or data on whether people were detected through passive or active case finding—a distinction that may better reflect disease severity than reported symptoms at screening [5]. Where countries have the capacity, a combination of microbiological, radiological, and clinical indicators would provide the most comprehensive indication of disease severity.

Currently, WHO TB incidence estimates do not distinguish aTB and sTB. While prevalence surveys suggest that around half of people with prevalent TB do not report symptoms, quantifying the role of symptoms in incidence is far more challenging, due to a lack of data to robustly inform such estimates and due to the extensive limitations of reported symptoms as an indicator of disease state. In any ongoing consideration of the role of symptoms in incidence estimates, it is essential that the results reflect the very substantial uncertainty that exists in TB natural history, especially in relation to symptomatology. Mathematical modeling of TB more generally (eg for burden estimation or to project the potential impact of intervention approaches) is likely to be more robust if models explicitly acknowledge the limitations of reported symptoms, for instance, through using more objective indicators of disease severity to define simulated disease states.

## CONCLUSIONS

The substantial proportion of people with prevalent TB who do not report symptoms means that the limitations of symptom screening need to be acknowledged in the development of TB prevention and care policies and research conducted into the potential yields of screening using symptoms versus alternative methods (eg chest X-ray). This does not mean that aTB and sTB are meaningful and distinct case definitions, however, or that reported symptoms should be used to inform public health surveillance, guide individual clinical management, or define disease states in models. We show that symptom reporting is heavily influenced by setting, context, and interviewers and that incidental TB symptoms are common, even in people with TB. Future policy development and research into the implications of the spectrum of TB disease should prioritize more objective severity metrics, alongside or in place of reported symptom status.
